# Cardiovascular Dysfunction Presenting as Autonomic Dysreflexia in a Patient with Spinal Cord Injury

**DOI:** 10.7759/cureus.1456

**Published:** 2017-07-10

**Authors:** Ahmed H Qavi, Salman Assad, Wardha Shabbir, Maryam Kundi, Maham Habib, Sumbal Babar, Mehr Zahid

**Affiliations:** 1 Department of Medicine, Mahroof Hospital; 2 Department of Medicine, Shifa International Hospital, Islamabad, Pakistan; 3 MD, Windsor University Medical School; 4 Pain Management, Institute of Advanced Medicine and Surgery, Philadelphia, Pa; 5 Department of Internal Medicine, Carthage Area Hospital, New York, USA; 6 Internal Medicine, University of Lahore, Lahore, Pakistan

**Keywords:** autonomic dysreflexia, spinal cord, hypertension, cardiovascular

## Abstract

Autonomic dysreflexia (AD) is a medical emergency that is characterized by hypertension as an autonomic response to noxious stimuli in patients with a history of spinal cord injury at the level of T6 or above. We present the case of a 31-year-old Caucasian male with a history of spinal cord injury at the level of C3-C4, with symptoms described as recurring episodes of hypertension with flushing and sweating above the level of the lesion for the past five to six years. His symptoms are triggered by bowel distention, excitement, a bumpy car ride, or a simple turning of the neck to the left. Physical examination and laboratory studies ruled out other possible differentials (e.g., migraines, pheochromocytoma). As a result, AD was diagnosed.

## Introduction

Autonomic dysreflexia (AD) is a condition that falls under the broad term "incomplete cord syndromes" and has variable neurological findings with a partial loss of sensory and motor function below the level of the lesion. A spinal cord injury (SCI) lower than T6 is less likely to cause this complication because an intact splanchnic innervation allows for the compensatory dilatation of the splanchnic vascular bed.

Several triggers have been recognized to cause these episodes, including bladder distension, urinary tract infections, bladder or kidney stones, urological procedures, such as the insertion of a urinary catheter, bowel distension caused by fecal impaction, hemorrhoids, and rectal irritation due to enema or manual evacuation. Ingrown toenails, tight clothing, and sexual intercourse may also trigger the attacks. Symptoms include a pounding headache (the most common symptom), cutis anserine (below the level of the injury), flushing (above the level of the injury), sweating (above the level of the injury), nasal congestion (above the level of the injury), red blotching of the skin (above the level of the injury), cold clammy skin (below the level of the injury), malaise, nausea, blurred vision, and restlessness. Signs include hypertension (more than 200/100 mmHg), slow pulse, and bradycardia (less than 60 beats/min).

Diagnostic clinical criteria include an increase in systolic blood pressure by at least 20-40 mmHg and any of the following symptoms: sweating, chills, cutis anserine, headache, and flushing. In these patients, the resting blood pressure is lower than that of people without a spinal cord injury (SCI).

## Case presentation

A 31-year-old male has experienced hot flashes for the past five to six years; this is associated with obvious erythema of the face and is accompanied by hypertensive episodes as high as 226/128 mmHg with no palpitations. In the past year, these spells had become more frequent with headaches in both temples. He also reports decreased peripheral vision during these episodes. Lying down seems to reduce the severity and duration of these spells. He often feels light-headed in the upright position and experiences vertigo while lying on his side, at times; this is always relieved by changing his position on the bed. His symptoms are relieved by sublingual medication, such as nifedipine, but the patient states that his episodes are brought on by excitement such as watching a football game, walking barefoot on hard surfaces, having a bumpy car ride, or making sudden head movements. He also reports heat intolerance and excessive sweating. The patient's past medical history was positive for SCI, after which he developed pain in the neck and lower back, with numbness and tingling in his upper extremities and pain radiating down his hip. Table 1 lists the common signs and symptoms of AD generally experienced by patients with SCI.

**Table 1:** Clinical Manifestations of AD in Patients with Spinal Cord Injuries AD: Autonomic Dysreflexia

Clinical Manifestations of AD in Patients with Spinal Cord Injuries
Moderate to severe headache
Flushing and piloerection above the injury
Dry and pale skin due to vasoconstriction below the level of injury
Blurred vision
Anxiety
Nasal congestion
Profuse sweating above the level of injury
Bradycardia
Cardiac arrhythmias, atrial fibrillation

Laboratory results revealed an increased excretion of epinephrine and dopamine but a normal output of norepinephrine. His epinephrine levels were increased by injections of intravenous glucagon, a response highly suggestive of the presence of pheochromocytoma. He also had prolonged spells of tachycardia induced by exercise despite taking propranolol, which was further suggestive of the production of large amounts of epinephrine, overriding the effects of propranolol.

**Figure 1:**
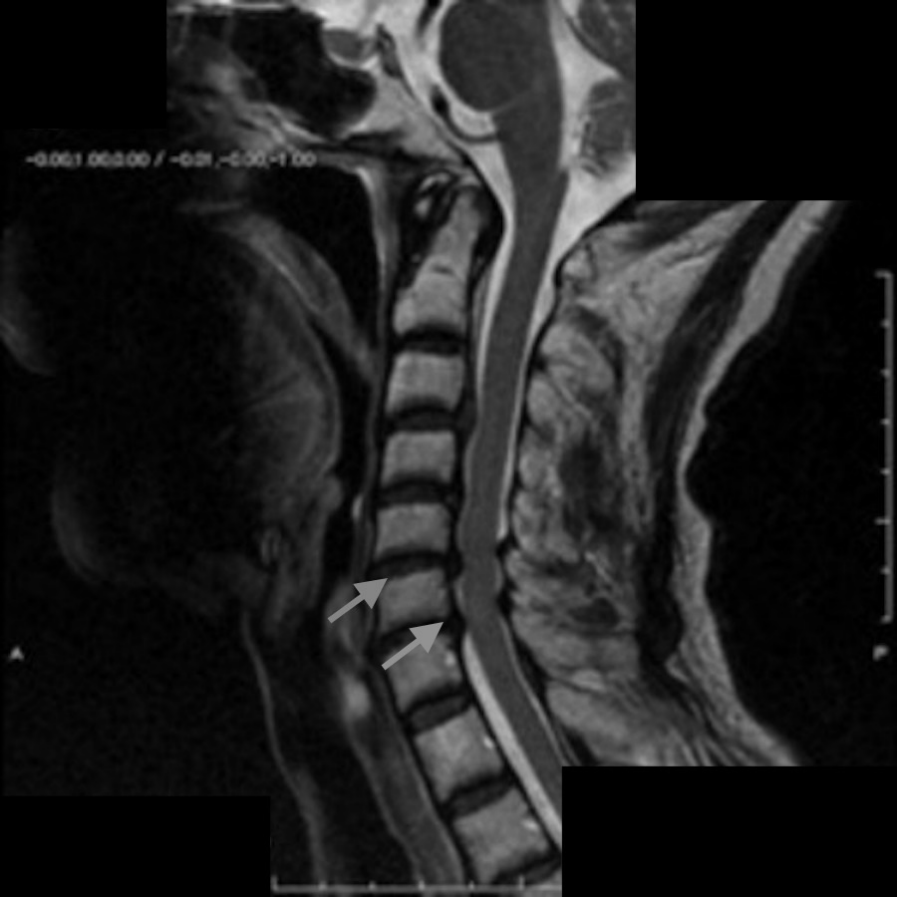
MRI showing dilated biliary ducts (white arrow) with a tapering of the distal common bile duct (yellow arrow) Cervical disc herniation at the level of C3–4 and C4–5 (gray arrows)

He underwent an evaluation for pheochromocytoma. An octreotide scan, a metaiodobenzylguanidine (MIBG) scan, and a magnetic resonance imaging (MRI) scan of the adrenal glands were all negative. After some time, the patient reported episodic anxiety and hypertension with a full bladder and immediate relief after voiding. He was sent for cystoscopy and urodynamic studies, which were all negative as well. Given his history of SCI, the patient underwent spinal cord imaging, which showed disk herniation at C3-C4 and C4-C5, as shown in Figure 1. After ruling out other possible etiologies, he was diagnosed with AD (a functional spinal cord disorder) in light of his symptoms appearing after trauma to the spinal cord.

## Discussion

Patients with SCI at the level of T6 or above are at risk of AD, which usually presents two to three months after the injury [1]. Our case had SCI at the level of L4/L5. One of the main characteristics of AD is paroxysmal hypertensive episodes that can result from any ascending sensory stimulus below the level of the injury [2]. The stimulus triggers the reflex activity of the sympathetic nervous system, and because of the isolation of the spinal cord from the vasomotor centers in the brain caused by the SCI, this leads to over-activity of the sympathetic ganglia [3].

Various substances, such as noradrenaline, cause severe vasoconstriction, leading to hypertension. The baroreceptors sense the rise in blood pressure via the aortic arch and carotid bodies, leading to compensatory bradycardia. The inhibitory activity of the central nervous system reaches the level of the neurogenic injury, resulting in parasympathetic activity (i.e., bradycardia, vasodilation) above the level of the lesion and sympathetic activity (i.e., vasoconstriction) prevails below the level of the lesion.

Our case is unusual because one of the main stimuli for our patient was simply turning his neck to the left. The stimulus was above the level of the spinal cord lesion, which is of particular importance, as in AD, the sensory stimulus ascends from below the level of the injury.

Milligan et al. highlighted the importance of family physicians knowing which patients with SCI are at risk of developing AD and familiarizing themselves with the symptoms associated with AD. This, in turn, helps diagnose and treat patients appropriately over time [4]. Our case displayed the typical signs and symptoms of AD, but because of the lack of familiarity with this complication, our patient was misdiagnosed several times. McGillivray et al. found that 22% of the patients with SCI who had reported symptoms of AD had no knowledge of their condition and had never heard of AD as being one of the complications of SCI [5].

AD is essentially an emergency situation; as a result, prompt diagnosis and treatment are essential for the patients’ wellbeing. The high blood pressure that develops during the hypertensive episodes can be detrimental and lead to cerebrovascular accidents and cardiovascular dysfunction. As a result, palliative treatment should be administered quickly [6]. Krassioukov et al. conducted a systematic review of 31 studies that evaluated the various measures taken to manage paroxysmal hypertensive episodes in SCI patients [7]. The initial steps should be nonpharmacological. Identifying, locating, and removing the stimulus should be the priority followed by the removal of any tight garments and positioning the patient upright. If the patient remains hypertensive, antihypertensive drugs should be administered promptly; these include prazosin (level 1 evidence) and nifedipine and prostaglandin E (level 2 evidence) [7]. Nitrates and captopril have also proven effective in terminating hypertensive episodes [8].

Bladder and bowel distension have been noted to be one of the most common stimuli that lead to hypertensive episodes in AD [9]. Rabchevsky et al. concluded that the administration of gabapentin to patients with SCI who are at risk of developing AD as a complication would suppress the noxious stimuli (bowel and bladder distension), resulting in the alleviation of AD [10]. Palliative treatment is well-known among physicians who are aware of AD being a complication of SCI above the level of T6 but preventative treatment has yet to be explored and employed [10].

## Conclusions

Since cases of AD can only be diagnosed based on physical presentation, emergency physicians and other healthcare workers should be thoroughly educated when it comes to the prompt management of patients presenting with signs and symptoms consistent with this condition. Increasing the awareness of AD among staff in emergency departments as well as educating patients and caretakers will be useful in preventing this complication in persons with SCI.
